# Do standards of care and early outcomes of periprosthetic fractures change during the COVID-19 pandemic? A multicentre study

**DOI:** 10.1186/s10195-021-00584-w

**Published:** 2021-06-14

**Authors:** Luigi Zagra, Rocco D’Apolito, Nicola Guindani, Giovanni Zatti, Fabrizio Rivera, Flavio Ravasi, Mario Mosconi, Alberto Momoli, Alessandro Massè, Massimo Franceschini, Fabio D’Angelo, Dante Dallari, Fabio Catani, Alessandro Casiraghi, Federico Bove, Claudio Carlo Castelli

**Affiliations:** 1 IRCCS Istituto Ortopedico Galeazzi, Via R. Galeazzi 4, 20161 Milan, Italy; 2 Department of Orthopaedic Surgery, ASST Papa Giovanni XXIII, Piazza OMS 1, 24127 Bergamo, Italy; 3 Department of Orthopaedic Surgery ASST Di Monza, Università Milano Bicocca, Via Pergolesi 33, 20900 Monza, Italy; 4 Department of Orthopaedic Surgery, Ospedale SS Annunziata, Via Ospedali 14, 12038 Savigliano, Italy; 5 Department of Orthopaedic Surgery, ASST Melegnano Martesana- Ospedale Di Vizzolo Predabissi, Via Pandina 1, 20077 Vizzolo Predabissi, Italy; 6 Department of Orthopaedic Surgery, IRCCS Policlinico San Matteo Di Pavia, Viale C. Golgi 19, 27100 Pavia, Italy; 7 Department of Orthopaedic Surgery, Ospedale San Bortolo, Viale F. Rodolfi 37, 36100 Vicenza, Italy; 8 Department of Orthopaedic Surgery, Ospedale Città Della Salute E Della Scienza Università Di Torino, Via G. Zuretti 29, 10126 Turin, Italy; 9 ASST Gaetano Pini- CTO, Piazza A. Ferrari 1, 20122 Milan, Italy; 10 Division of Orthopaedics and Traumatology, ASST Dei Sette Laghi, Department of Biotechnology and Life Sciences (DBSV), University of Insubria, Viale L. Borri 57, 21100 Varese, Italy; 11 Reconstructive Orthopaedic Surgery and Innovative Techniques-Musculoskeletal Tissue Bank IRCCS Istituto Ortopedico Rizzoli, via G.C. Pupilli 1, 40136 Bologna, Italy; 12 Department of Orthopaedic Surgery, Policlinico Universitario Di Modena, Via del Pozzo 71, 41124 Modena, Italy; 13 Department of Orthopaedic Surgery, ASST Degli Spedali Civili Di Brescia, Piazzale Spedali Civili 1, 25123 Brescia, Italy; 14 Department of Orthopaedic Surgery, ASST Grande Ospedale Metropolitano Niguarda, Piazza Ospedale Maggiore 3, 20162 Milan, Italy

**Keywords:** Periprosthetic fractures, Coronavirus, COVID-19, Joint arthroplasty, Revision, Osteosynthesis, Hip, Knee, Femur, Complications

## Abstract

**Background:**

Periprosthetic fractures (PPFs) are a growing matter for orthopaedic surgeons, and patients with PPFs may represent a frail target in the case of severe acute respiratory syndrome coronavirus 2 (SARS-CoV-2) infection. The purpose of this study is to investigate whether hospital reorganisations during the most severe phase of the SARS-CoV-2 pandemic affected standards of care and early outcomes of patients treated for PPFs in Northern Italy.

**Materials and methods:**

Data were retrieved from a multicentre retrospective orthopaedics and traumatology database, including 14 hospitals. The following parameters were studied: demographics, results of nasopharyngeal swabs, prevalence of coronavirus disease 2019 (COVID-19), comorbidities, general health status (EQ-5D-5L Score), frailty (Clinical Frailty Scale, CFS), pain (visual analogue scale, VAS), anaesthesiologic risk (American Society of Anaesthesiology Score, ASA Score), classification (unified classification system, UCS), type of operation and anaesthesia, in-hospital and early complications (Clavien–Dindo Classification, CDC), and length of stay (LOS). Data were analysed by means of descriptive statistics. Out of 1390 patients treated for any reason, 38 PPFs were included.

**Results:**

Median age was 81 years (range 70–96 years). Twenty-three patients (60.5%) were swabbed on admission, and two of them (5.3%) tested positive; in three patients (7.9%), the diagnosis of COVID-19 was established on a clinical and radiological basis. Two more patients tested positive post-operatively, and one of them died due to COVID-19. Thirty-three patients (86.8%) presented a proximal femoral PPF. Median ASA Score was 3 (range, 1–4), median VAS score on admission was 3 (range, 0–6), median CFS was 4 (range, 1–8), median EQ-5D-5L Score was 3 in each one of the categories (range, 1–5). Twenty-three patients (60.5%) developed post-operative complications, and median CDC grade was 3 (range, 1–5). The median LOS was 12.8 days (range 2–36 days), and 21 patients (55.3%) were discharged home.

**Conclusions:**

The incidence of PPFs did not seem to change during the lockdown. Patients were mainly elderly with comorbidities, and complications were frequently recorded post-operatively. Despite the difficult period for the healthcare system, hospitals were able to provide effective conventional surgical treatments for PPFs, which were not negatively influenced by the reorganisation. Continued efforts are required to optimise the treatment of these frail patients in the period of the pandemic, minimising the risk of contamination, and to limit the incidence of PPFs in the future.

**Level of evidence:**

IV.

## Introduction

Coronavirus disease 2019 (COVID-19) has gradually hit the entire world; after the first reports from Wuhan [1], the capital of Hubei province in China, Italy was the first country facing this emergency early in 2020. At the end of January, the first two cases of infection in Italy were a Chinese couple vacationing in Rome, whereas the first Italian citizen testing positive for the severe acute respiratory syndrome coronavirus 2 virus (SARS-CoV-2) was a young man in the south of Lombardy, a region of Northern Italy, on 21 February. From the day after, the National Government announced the quarantine of people in several municipalities of Lombardy and Veneto (a neighbouring region), in the so-called red zones (meaning the block of entrances and exits from these areas). On 7 March, Lombardy and 14 other provinces of Northern Italy were declared red zones. Four days later, considering the spread of the virus, a national lockdown was introduced [2]. In parallel, the national public health system [Servizio Sanitario Nazionale (SSN)] was dramatically affected by the rapid pandemic spread. Most general hospitals were overwhelmed with SARS-CoV-2 patients, and consequently many departments were converted to COVID-19 care centres. According to the regional authorities, elective surgeries were gradually suspended, and non-deferrable orthopaedics and trauma cases were referred to selected centres, designated as either minor or major trauma hubs [3].

SARS-CoV-2 infection may range from asymptomatic or mild to severe or critical disease [4]. It has been shown that epidemiological characteristics may affect the prognosis of COVID-19 [5]. Moreover, literature data suggest that COVID-19 affects older patients and those with comorbidities most severely [6, 7]. Patients with periprosthetic fractures (PPFs) are often old and with comorbidities. PPFs are less frequent than other types of bone fractures, but increasing numbers of joint arthroplasties performed each year, with projection continuously rising for the future [8], at the same pace as longer life expectancies and prevalence of osteoporosis, contribute to an expanding number of PPFs [9]. Surgeons facing these pathologies have to solve several problems, from the correct diagnosis and classification of the fracture to the assessment of implant stability, both conditioning the type of treatment [10]. These procedures are often complex, and patients are at considerable risk of medical and surgical morbidity [9]. The surgical complexity together with the fragility of these patients increases costs, but also length of stay (LOS) [11]. Hence, patients with PPFs might represent a group at increased risk of developing clinical symptoms and a severe course of COVID-19 when infected by SARS-CoV-2.

Most of the studies published in this difficult period have analysed fractures in general, with few studies focusing on hip fracture management and COVID-19 implications for this particular group of patients [12, 13]. However, thus far, no one has reported specifically about PPFs. The purpose of this multicentre retrospective study is to investigate whether hospital reorganisations during the most severe phase of SARS-CoV-2 pandemic affected standards of care and early outcomes of patients treated for PPFs in Northern Italy.

## Materials and methods

Data from 14 hospitals located in Northern Italy were collected during the lockdown (from 9 March to 4 May 2020); during this “red” phase, SARS-CoV-2 was spreading widely and was not contained [14]. The reference centre (Bergamo) gathered all the data from the other centres in aggregated and anonymous form. The study was approved by the institutional review board of the reference centre (Bergamo, Number 31_21). All procedures were performed in accordance with the ethical standards of the institutional committee and the tenets of the 1964 Helsinki Declaration and its later amendments. The centres involved in the study were the following: Papa Giovanni XXIII Hospital of Bergamo, IRCCS Rizzoli Orthopaedic Institute of Bologna, Civilian Hospital of Brescia, Niguarda Hospital of Milan, IRCCS Galeazzi Orthopaedic Institute of Milan, Teaching Hospital of Modena, San Gerardo Hospital of Monza, IRCCS San Matteo Teaching Hospital of Pavia, SS Annunziata Hospital of Savigliano, Teaching Hospital of Turin, Teaching Hospital of Varese, and San Bortolo Hospital of Vicenza. All patients admitted to the emergency departments of each hospital with a diagnosis of PPF and scheduled for surgical treatment in the 2-month period were included. Among the centres, there were nine tertiary care hospitals, three orthopaedic clinics and two secondary care hospitals.

During the study period, all the hospitals established protocols and precautions to limit the spread of the virus [3]. The entrances to the hospitals were forbidden to visitors. Dedicated wards were created to admit COVID-19 patients, and distinct operating rooms were reserved to these patients in theatre blocks. These rooms were marked with clearly visible door sign, and specific pathways were created to connect the wards with the operating rooms with the purpose of preventing contamination of other “clear” pathways for employees and SARS-CoV-2-negative patients. All personnel were specifically trained to don, doff and dispose of personal protective equipment including masks, eye protection, double gloves, gowns, suits and caps, both for the operating rooms and for the wards dedicated to COVID-19 patients [15, 16]. Ways in and out of these spaces were separated for the staff.

The collected data of interest were the following: demographics (age and gender), results of reverse-transcription polymerase chain reaction (PCR) nasopharyngeal swab test (positive or negative) [17, 18], prevalence of COVID-19 on admission and discharge (according to the clinical, diagnostic imaging, laboratory and epidemiological criteria of the European Centre for Disease Prevention and Control [19]), comorbidities, general health status (EQ-5D-5L Score [20]), frailty (Clinical Frailty Scale, CFS [21]), pain (visual analogue scale, VAS [22]) and anaesthesiologic risk (American Society of Anaesthesiology, ASA Score [23]).

PPFs were classified according to the Unified Classification System (UCS) [24]. The type of operation was noted (osteosynthesis and/or revision) along with the type of anaesthesia. In-hospital and early complications were recorded and graded according to the Clavien–Dindo Classification (CDC) [25]. LOS and type of discharge (at home/to intensive rehabilitation centre/extended care unit) were part of the collected data, along with medical records from clinical examination or phone/telemedicine call at 1-month follow-up.

Sample distributions were tested for normality with the Kolmogorov–Smirnov test. Accordingly, data were described as non-parametric. Statistical analyses were computed using Microsoft Excel v. 16.0 (Microsoft Corporation, Redmond, WA).

## Results

A total of 1390 patients were admitted for acute care in orthopaedics and trauma during the observed period, 38 of which (2.7%) had PPF. Of these, 12 (31.5%) were males and 26 (68.5%) females, with a median age of 81 years (range, 70–96 years). A screening for SARS-CoV-2 by means of swab test was performed in 23 out of 38 patients (60.5%) on admission, and 2 out of 38 (5.3%) tested positive; in three out of 38 patients (7.9%), the diagnosis of COVID-19 was established on a clinical basis. Two of these five patients had the diagnosis pre-operatively, whereas in the remaining three cases the diagnosis was done post-operatively.

Of 38 patients, 33 (86.8%) presented a femoral PPF – 7 (18.4%) regarding the distal femur, 1 of which occurred in a patient with a knee spacer. Three patients (7.9%) had an acetabular PPF, one patient (2.6%) tibial PPF, and one patient (2.6%) humeral fracture. The UCS classifications of the fractures are reported in Table 1.

**Table 1:** Types of fractures according to the Unified Classification System

UCS	*n* of patients
II1.B1	1
IV3.B1	13
IV3.B2	10
IV3.B3	1
IV3.C	2
IV4.C	1
IV6.B1	2
IV6.B2	1
V3.B1	2
V3.B2	2
V3.C	2
V3.D	1

The median ASA Score was 3 (range 1–4). Sixteen patients (42.1%) had a score of 2, 17 patients (44.7%) had a score of 3, 4 patients (10.5%) had a score of 4, and 1 patient (2.6%) had a score of 1. The median VAS score on admission was 3 (range 0–6) (Table 2), whereas the median CFS was 4 (range 1–8) (Table 3); the median EQ-5D-5L Score was 3 in each of the five categories (range 1–5) (Table 4).

**Table 2:** Pain on admission according visual analogue scale

VAS	*n* of patients
0	2
1	5
2	6
3	10
4	6
5	4
6	5
7	0
8	0
9	0
10	0

**Table 3:** Clinical Frailty Scale

CFS	*n* of patients
0	0
1	1
2	2
3	8
4	9
5	8
6	2
7	6
8	2
9	0

**Table 4:** General health status

EQ-5D-5L Score					
Score	1	2	3	4	5
Mobility	1 (2.6%)	3 (7.9%)	7 (18.4%)	16 (42.1%)	11 (28.9%)
Self-care	6 (15.8%)	10 (26.3%)	4 (10.5%)	8 (21.1%)	10 (26.3%)
Usual activities	9 (23.7%)	9 (23.7%)	7 (18.4%)	11 (28.9%)	2 (5.3%)
Pain/discomfort	7 (18.4%)	9 (23.7%)	12 (31.6%)	10 (26.3%)	0
Anxiety/depression	8 (21.1%)	10 (26.3%)	10 (26.3%)	9 (23.7%)	1 (2.6%)

The most frequent comorbidities were hypertension (16 patients, 42.1%) and diabetes (10 patients, 26.3%) (Table 5).

**Table 5:** Main comorbidities in the study population

Comorbidities	*n* of patients
Hypertension	16
Diabetes	10
Vascular disorders	5
Endocrinology disorders	6
AF	3
Cancer	2
COPD	3
Mental disorders or dementia	5
Rheumatic pathologies	2
Hypercholesterolemia	2
Hepatitis	2
Blood diseases	2

*AF* Atrial fibrillation; *COPD* chronic obstructive pulmonary disease

Regarding surgical procedures, 19 patients (50%) underwent fracture reduction and osteosynthesis with component retention, 12 (31.6%) underwent component revision and osteosynthesis, and 7 (18.4%) underwent component revision alone. Twenty patients (52.6%) received spinal anaesthesia, whereas ten patients (26.3%) underwent general anaesthesia. Among the others, five (13.2%) patients received spinal anaesthesia plus peripheral nerve blocks, one (2.6%) general plus peripheral block, one (2.6%) blended anaesthesia (general plus spinal), and one (2.6%) peripheral block alone.

Twenty-three patients (60.5%) developed post-operative complications. The median CDC grade was 3 (range 1–5) (Table 6).

**Table 6:** Grades of complications according to Clavien–Dindo Classification

CDC	*n* of patients
0	2
1	3
2	6
3	16
4	6
5	5

The most frequent in-hospital complication was anaemia, which affected 18 patients (47.4%). Two patients (5.3%) developed non-COVID-related pneumonia, one patient (2.6%) urinary tract infection, one (2.6%) intracerebral haemorrhage, and one (2.6%) dysuria. With regard to COVID-19, one patient (2.6%), with negative swab and asymptomatic on admission, tested positive post-operatively, developed a worsening clinical picture during hospital stay and eventually died. After discharge, two patients (5.3%) developed anaemia, one patient (2.6%) dyspnoea and non-COVID-related pneumonia, one patient (2.6%) transient ischemic attack, one patient (2.6%) urinary tract infection, one patient (2.6%) total hip arthroplasty dislocation, and one patient (2.6%) wound dehiscence. Regarding COVID-19, one patient (2.6%) tested positive after discharge and developed a mild syndrome with fever requiring hospital admission before complete recovery.

The median LOS was 12.8 days (range, 2–36 days). Twenty-one patients (55.3%) were discharged home and 17 (44.7%) to intensive rehabilitation centres or extended care units.

## Discussion

The COVID-19 pandemic has caused a huge number of hospitalisations and deaths, especially in elderly and patients with comorbidities, and is importantly affecting orthopaedic practice [3]. The incidence of PPFs, mostly affecting older patients with multiple medical pathologies as well, is rising and presents a significant clinical and economic burden [26–29]. Hence, PPFs in the COVID-19 era might have been negatively affected by the pandemic, although no data have shown this correlation thus far. This study aimed to investigate standards of care and early outcomes of patients treated for PPFs, reporting demographic characteristics, treatments, and in-hospital and early complications in 14 centres of Northern Italy during the first wave of COVID-19 pandemic. In the study period, 38 patients were admitted in emergency with a diagnosis of PPF (3% of all admissions). As expected, most patients were elderly, with the majority being women. Many patients presented considerable medical comorbidities, with all but one having an ASA Score of 2 or higher, and almost half having a score of 3. The proximal femur was the segment most frequently involved, and the leading procedure was osteosynthesis, which in one-third of the patients was part of a component revision procedure. Notably, the proportion of complications was high, with three patients out of five developing some. One of the two patients testing positive for COVID-19 post-operatively developed a severe and progressively worsening clinical picture and eventually died. The median LOS exceeded 10 days, and almost half of patients was discharged to intensive or extended care units.

Despite the study period coinciding with a lockdown, the proportion of admissions for PPFs (3%) seemed to be similar to that observed in 2019 in our centres and that previously reported in literature [30]. Expanding indications of total joint arthroplasties (TJAs) both in younger and adults, along with rising numbers of procedures performed per years, growing number of cementless fixation, increasing life expectancy and growing prevalence of osteoporosis, are all factors contributing to the increase of periprosthetic fractures worldwide [26, 31, 32]. Because frail elderly patients with osteoporosis represent the patients most at risk, the mechanism of injury is often a low-energy fall from sitting or standing heights [33], and this kind of fall often happens at home. Therefore, it is reasonable that restrictions concerning social life have not slowed down this trend too much, in this selected population.

The pandemic put a strain on the healthcare system, with considerable human and structural resources used to face this emergency. In most of the hospitals, orthopaedic surgeons were redirected to COVID units, and 14% of the staff was daily occupied in these activities. Moreover, despite precautions, some of the staff tested positive during the study period, increasing the workload for the personnel. Among the centres, 11% of orthopaedic surgeons developed COVID-19, without long-term consequences. Nevertheless, for patients needing surgical treatment, including those suffering from PPFs, an effort was made to provide the best options to everyone despite the ongoing situation. These circumstances did not influence the choice of surgical treatments, which was adapted to the specific case instead, as it would have been done in a non-pandemic scenario.

SARS-CoV-2 infection may cause a broad spectrum of clinical manifestations, ranging from totally asymptomatic infections to severe COVID-19 cases that may have a poor prognosis. It has been shown that patients older than 65 years may have a greater risk of developing critical or mortal COVID-19, and comorbidities such as hypertension, diabetes, cardiovascular disease and respiratory diseases may also greatly affect the prognosis of the disease [6]. The youngest of our patients was 70 years, and most of them presented comorbidities, frequently hypertension and diabetes. Moreover, COVID-19-positive patients with hip fractures, who may be similar to those with periprosthetic fractures except for the fact that the latter usually undergo a more invasive procedure, have shown a higher 30-day mortality rate compared with non-COVID-19 infected cases [7], and, more broadly, a meta-analysis revealed a very high global rate of post-operative mortality among COVID-19 patients of 20% and a post-operative intensive care unit (ICU) admission rate of 15% [34]. The patient’s immune function has been reported as a major determinant of the disease’s severity [35], and surgery may cause both an immediate impairment of immune function and an early systemic inflammatory response [36]. Thus, surgeons should pay close attention to patients suffering from PPFs and undergoing surgical treatments in this characteristic period, because SARS-CoV-2 infection may have a detrimental course in these cases.

The average LOS of our patients (13 days) is similar to that reported recently in data from a national study performed in the UK in a 3-year timeframe [37], which showed a median acute LOS of 14 nights. This length might have been influenced both by the fact that many rehabilitation centres were closed during the pandemic and by the willingness of patients’ relatives to shorten the length of stay as much as possible in this period to reduce the risks of transmission. Therefore, 55% of patients were directly discharged at home.

COVID-19 is causing massive employment of financial resources for healthcare systems, and economic impact of PPFs is even more important in this period, in which this “epidemic” adds to the worst health emergency of our time. In this regard, it is worth remembering that PPF rates may range from 0.1% to 18% for hip, 0.3% to 5.5% for knee and 0.5% to 3% for shoulder arthroplasties [38]; that 0.8–1.1% of total hip arthroplasty (THA) and 0.5–1.1% of total knee arthroplasty (TKA) patients will experience a periprosthetic fracture in the first 5 years [26]; and that 5 years post-operation, fracture is the main cause of revision for THA in patients older than 75 years, with a significant increase in the case of cementless implants [39]. So, the rising incidence, costs related to the surgical procedure, complications and hospitalisation should lead healthcare settings and surgeons to keep their attention on this pathology during this difficult period. Moreover, PPF treatment requires skilled surgical teams, more expensive and complex implants, availability of revision and fixation systems, information on previous implants, and sometimes bone grafts. All these requirements might be extremely difficult to meet during a pandemic.

Our study has several limitations. Firstly, it is likely that heterogeneity influenced our findings, both because different joints were included and because of the multicentre design of the study. However, most patients suffered from hip PPF, and influence of other joints, presumably implying less morbidity of the procedures with an overestimation of the outcomes, is minimal. Moreover, different surgeons from several institutions were involved, but all of them are experienced in the treatment of such pathology, sharing decision-making about the procedure based on PPF classifications. The multicentre nature of the study also represents a strength, because it would have not been possible to collect this number of cases in a limited period otherwise. Secondly, the short follow-up period probably caused an underestimation of the mortality rate; longer-term follow-up will be needed to assess morbidity and mortality beyond the early post-operative period. However, all the patients underwent at least one medical evaluation at 1-month follow-up. In addition, both the small number of confirmed cases of COVID-19 (with positive swab) and patients with a clinical and radiological diagnosis limit our conclusions about the effect of COVID-19 on patients with PPFs patients. In particular, the only two patients with a pre-operative diagnosis presented a mild clinical picture and underwent osteosynthesis. It is likely that the type of revision might be influenced by COVID-19, but no conclusions may be drawn on this topic on the basis of our data. In the same way, it is possible that more-severe clinical pictures of COVID-19 preclude any treatment for PPFs at all. We warn the reader that SARS-CoV-2 infections might have been underestimated, because at the beginning of the pandemic not all patients were tested. Indeed, it is possible that a certain number of asymptomatic patients have been included in this population. Nevertheless, this is a picture of the “real life” scenario occurring during a unique period of the COVID-19 outbreak in one of the first and most affected areas around the world, and to the best of our knowledge, this is the largest cohort of patients treated for PPFs during the COVID-19 pandemic reported to date.

## Conclusions

This study showed that there was no evident change in the incidence of PPFs following social restrictions imposed by the lockdown period during the first wave of the COVID-19 pandemic. In the setting of an emergency, despite a substantial number of resources redirected to handle the pandemic, hospitals were able to provide standard care for PPFs as before. Considering social restrictions, causing a decrease of major trauma from road accidents and work-related injuries, along with interruptions in elective surgeries, PPFs represented the most demanding surgeries performed in orthopaedic and trauma units in the study period. Our data confirm that these patients often present complex clinical pictures to be managed and stabilised before surgery, which may frequently involve implant revision and may entail a considerable rate of complications. In such patients, COVID-19 might assume a life-threatening course. A cautious pre-operative, intra-operative and post-operative management, along with a tailored surgical treatment, is of paramount importance these days more than ever to minimise the risk of SARS-CoV-2 contamination.

## Data Availability

All data generated or analysed during this study are included in this published article.

## Abbreviations

PPF: Periprosthetic fracture
SARS-CoV-2: Severe acute respiratory syndrome coronavirus 2
COVID-19: Coronavirus disease 2019
CFS: Clinical Frailty Scale
VAS: Visual analogue scale
ASA: American Society of Anaesthesiology
UCS: Unified classification system
CDC: Clavien–Dindo Classification
LOS: Length of stay
SSN: Servizio Santiario Nazionale
TJA: Total joint arthroplasty
